# Computer Aided Full Arch Restoration by Means of One-Piece Implants and Stackable Guide: A Technical Note

**DOI:** 10.3390/dj11110256

**Published:** 2023-11-01

**Authors:** Mattia Manfredini, Pier Paolo Poli, Carlo Maiorana, Federica Eugenia Salina, Marco Tandurella, Mario Beretta

**Affiliations:** 1 Department of Biomedical, Surgical and Dental Sciences, University of Milan, 20122 Milan, Italy; mattia.manfredini@unimi.it (M.M.); carlo.maiorana@unimi.it (C.M.); federicaeugenia.salina@studenti.unimi.it (F.E.S.); marco.tandurella@unimi.it (M.T.); mario.beretta@unimi.it (M.B.); 2 Implant Center for Edentulism and Jawbone Atrophies, Maxillofacial Surgery and Dental Unit, Fondazione IRCCS Cà Granda Ospedale Maggiore Policlinico, 20122 Milan, Italy

**Keywords:** digital implantology, computer-guided implant surgery, digital workflows, full-arch immediate loading, surgical template

## Abstract

This technical note aims to present a recently developed computer-guided protocol characterized by titanium-reinforced stackable surgical guides during post-extractive implant placement and subsequent immediate loading. A full maxillary edentulism was rehabilitated with one-piece implants, starting from a pre-existing removable denture. 3D digital scans of the removable denture and upper and lower arches were performed. On this basis, a prototype with ideal esthetic and functional outcomes was realized and replicated into a custom-made radiological stent with markers. The superimposition of STL and DICOM files allowed virtual planning of one-piece implants in the ideal prosthetically driven position. The stackable guides, composed of a fixed base template and additional removable components, were then realized. The fixed template, initially secured with anchor pins to the bone, was no longer removed. The removable components, which were screwed to the base template, were used to perform implant surgery and immediate prosthetic loading. No surgical complications occurred, the implants achieved a minimum insertion torque of 35 Ncm, and immediate prosthetic loading was performed. The base template allowed for the maintenance of a fixed reference during the entire workflow, improving the transition between the digital project, the surgical procedure, and the prosthetic rehabilitation.

## 1. Introduction

Computer-assisted implantology was introduced more than 25 years ago with the aim of optimizing implant positions according to the prosthetic project [1]. Furthermore, implant planning, supported by cone-beam computed tomography (CBCT), helps prevent intraoperative complications such as nerve injuries, sinus perforations, fenestrations, or dehiscences [2]. Additionally, the need to consider crucial factors, such as inter-implant and tooth-to-implant distances, implant depth, and position relative to the cortical plates, has made virtual planning an important instrument for achieving satisfactory outcomes [3,4]. Guided implantology is made possible through surgical templates that allow implant insertion in the planned position. Different types of surgical templates are available and categorized according to their stabilization to intraoral structures. Tooth-supported surgical guides are stabilized on remaining teeth, bone-supported guides are anchored to the bone via bone screws or pins, while mucosa-supported templates are positioned on the gingiva [5]. The latter are mostly used in flapless procedures, where implants can be inserted without elevating mucoperiosteal flaps. In general, a flapless approach simplifies the surgical phase, decreases the invasiveness of the surgery, minimizes pain, preserves blood circulation, and prevents periosteum damage [6,7,8,9]. This is particularly true in patients with appropriate bone volume and keratinized gingiva, as these conditions are ideal for a flapless technic [10]. Conversely, the elevation of a mucoperiosteal flap is indicated when hard and soft tissue deficiencies are present. In this way, bone regeneration and/or soft tissue optimization can be conducted simultaneously with implantation according to the prosthetic project.

Moreover, in order to obtain ideal esthetic and functional results, especially in the anterior sectors, prosthetic design should be considered at every surgical stage.

Stackable guides consist of a fixed base and removable components. This feature simplifies the supervision of all surgical and prosthetic steps. Furthermore, the stackable structure allows for the management of hard and soft tissues without removing the fixed base.

Accurate implant placement is crucial in case of immediate loading protocols, so that a prefabricated prosthesis can be connected to the implants immediately after the surgery, avoiding the need for intraoperative impressions [11]. Unfortunately, many cumulative errors can lead to positioning deviations throughout the entire workflow. Some of these may arise during the surgical procedure while positioning and stabilizing the surgical stent [12]. In this regard, stackable surgical guides with fixed references may improve the accuracy of implant placement and immediate loading [13]. In this way, operative time and patient discomfort are decreased. Moreover, eating capability, phonetics, aesthetics, and comfort are immediately restored, allowing patients to return to their normal activities in a short amount of time. It is also noteworthy that immediate implant loading in full-arch restorations is associated with good success rates in both arches, making this procedure predictable and reliable [14,15,16,17].

In addition to modular templates, one-piece implants combined with computer-guided workflows offer multiple advantages. The absence of an internal connection prevents bacterial infiltration and reduces prosthetic complications. Furthermore, a reduction in the number of prosthetic steps is achieved. On the other hand, precise digital planning, in order to obtain satisfactory primary stability and a correct position of one-piece implants, is mandatory [18]. In this context, this case aims to present a newly developed computer-guided protocol characterized by titanium-reinforced stackable surgical guides for one-piece implant placement and subsequent immediate loading.

## 2. Materials and Methods

**Figure 1:**
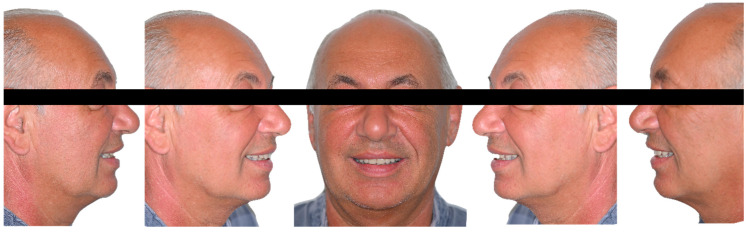
Extraoral photos with the maxillary removable denture.

This clinical report is included in an ongoing prospective study conducted in accordance with the ethical principles for medical research involving human subjects outlined in the World Medical Association Declaration of Helsinki, and was additionally approved by the local ethics committee (Fondazione IRCCS Cà Granda Ospedale Maggiore Policlinico, Milan area 2, registration ID #0002693-U). A 63-year-old male patient in good general health (ASA 1) presented with maxillary complete edentulism and rehabilitated with a removable denture (Figure 1). After discussing the possible therapeutic alternatives with the patient, an implant-supported rehabilitation with 6 one-piece implants was proposed and accepted. A digital workflow was used to facilitate planning and accomplish fully guided implant placement and immediate restoration in a single surgical session. The use of CBCT, photographs, digitalized models, intra-oral scanning, and interactive treatment planning software allowed for accurate pre-surgical planning, the fabrication of an innovative design for full-template guidance, and the management of the provisional milled PMMA (polymethylmethacrylate) titanium-reinforced restoration.

### 2.1. Surgical Planning

**Figure 2:**
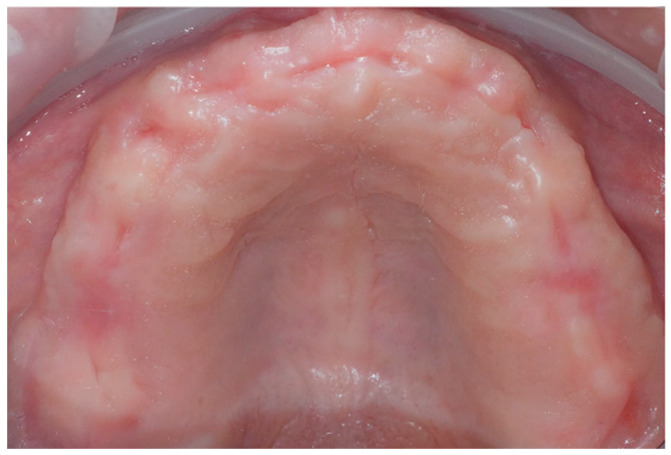
Intraoral view.

The patient presented a correct vertical dimension that was used as a reference during rehabilitation. Digital 3D scans of the complete removable denture worn by the patient (both the inner and outer parts) and the upper and lower arches were made with an intraoral scanner (CS 3600, Carestream Dental, Carestream Health, Atlanta, GA, USA). Intraoral and extraoral photographs were taken to create a customized virtual diagnostic wax-up, which was designed with dental computer-aided design (CAD) software (Exocad 3.0 DentalCAD, exocad Gmb, Darmstadt, Germany) (Figure 1 and Figure 2).

**Figure 3:**
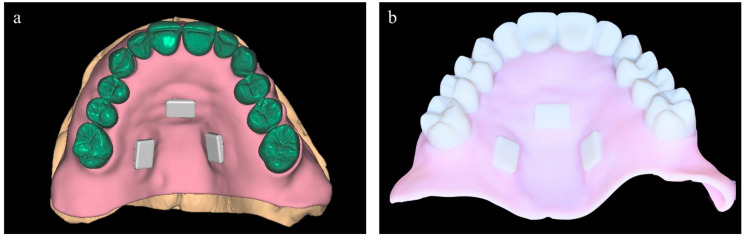
Digital planning of the radiological stent. (**a**) Virtual project. (**b**) Radiological stent equipped with radiopaque markers.

During the design of the diagnostic waxing, modifications of the complete removable denture were required to achieve optimal esthetic outcomes. A prototype, based on the digital waxing, was printed, which allowed for an additional intraoral assessment and also served as a radiographic stent, equipped with custom radiopaque markers (Figure 3). The prototype was created using printed resin (Clear MED610TM; Stratasys, Edina, MN, USA) with a 3D printer (J5 DentaJet; Stratasys, Edina, MN, USA).

A CBCT scan was performed to evaluate the anatomy of the residual bone (Carestream cs 9300, Rochester, New York, NY, USA). The standard triangulation language (STL) files obtained from the digital scan were aligned with the digital imaging and communication in medicine (DICOM) data retrieved from the CBCT scan. The superimposition was obtained by means of a best-fit adaptation algorithm that involved matching recognizable surfaces in both STL and DICOM datasets (RealGuide; 3DIEMME, Figino Serenza, Italy). In this case, the custom markers equipped in the radiological stent consisted of recognizable geometric shapes that the software was able to identify in both DICOM and STL files, allowing for the superimposition of radiological and digital scans.

**Figure 4:**
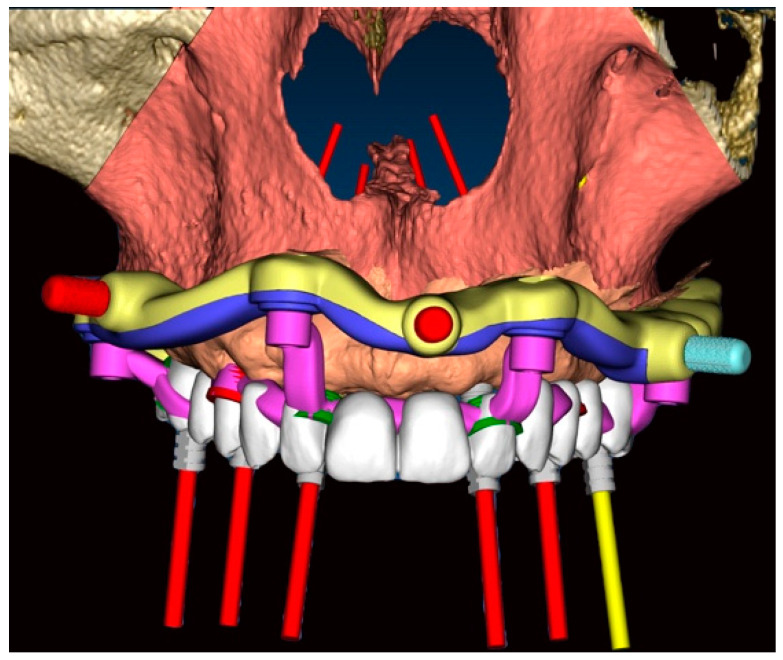
Virtual planning of ideal implant position: digital wax-up, base template (yellow and blue) with sleeves for anchor pins (red), and implant guide (violet).

**Figure 5:**
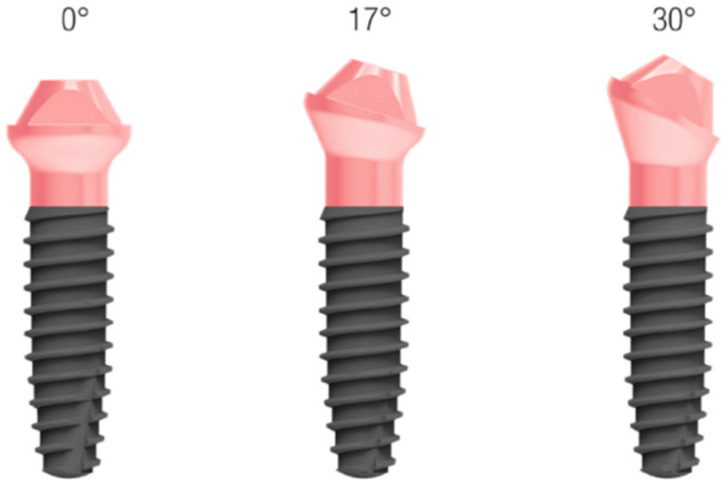
The one-piece implant with integrated MUA. 3 inclinations, 0°–17°–30°.

The ideal prosthetically driven position of one-piece implants was determined through virtual planning performed using dedicated software (RealGuide 5.2; 3DIEMME, Como, Italy) based on a digital waxing (Figure 4). Thus, six pre-angulated one-piece implants (FIXO; Oxy Implant Dental System, Biomec, Colico, Italy) were planned in the maxilla (Figure 5), as detailed below:

- 16: Oxy Implant FIXO Short 30° 4 × 10 mm
- 14: Oxy Implant FIXO Short 17° 4 × 10 mm
- 12: Oxy Implant FIXO Mini 17° 3.5 × 11.5 mm
- 22: Oxy Implant FIXO Mini 17° 3.5 × 13 mm
- 24: Oxy Implant FIXO Short 17° 4 × 11.5 mm
- 26: Oxy Implant FIXO Short 17° 4 × 8.5 mm

**Figure 6:**
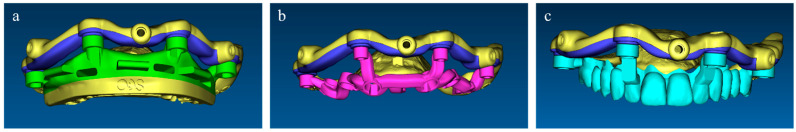
Project of stackable guide. (**a**) Base template (yellow and blue) with positioning template (green and yellow). (**b**) Base template and implant placement template (violet). (**c**) Base template and provisional prosthesis.

**Figure 7:**
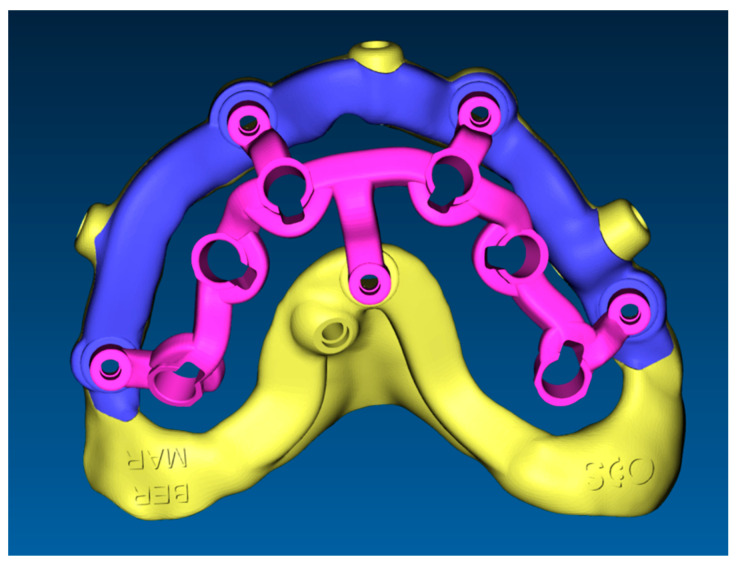
Occlusal view of the base template (yellow and blue) and implant placement template (violet). Details of notches in the sleeves for guided implant position.

The stackable guide (Figure 6), fabricated on the basis of the position of the implants in the digital project, was characterized by the following components:

- Fixed component or base template: printed resin (Clear MED610™; Stratasys, Edina, MN, USA), reinforced with milled titanium grade 5 (SINERGIA DISK Ti; Nobil Metal, Bergamo, Italy).
- Removable components, screwed to the base template, consisting of:
  - Positioning template: printed resin (Clear MED610™; Stratasys, Edina, MN, USA);
  - Implant placement template (Figure 7): milled titanium grade 5 (SINERGIA DISK Ti; Nobil Metal, Bergamo, Italy);
  - Provisional prosthesis: milled PMMA (Multilayer PMMA Disc; Dentsply Sirona, Verona, Italy) and a milled titanium grade 5 core (SINERGIA DISK Ti; Nobil Metal, Bergamo, Italy.

A specific miller (XD182; Faimond, Vicenza, Italy) was used to mill the titanium and PMMA in the base template, implant placement template, and provisional prosthesis. A 3D Printer (J5 DentaJet; Stratasys, Edina, MN, USA) was used to print the resin-made base template and positioning template.

### 2.2. Surgery

**Figure 8:**
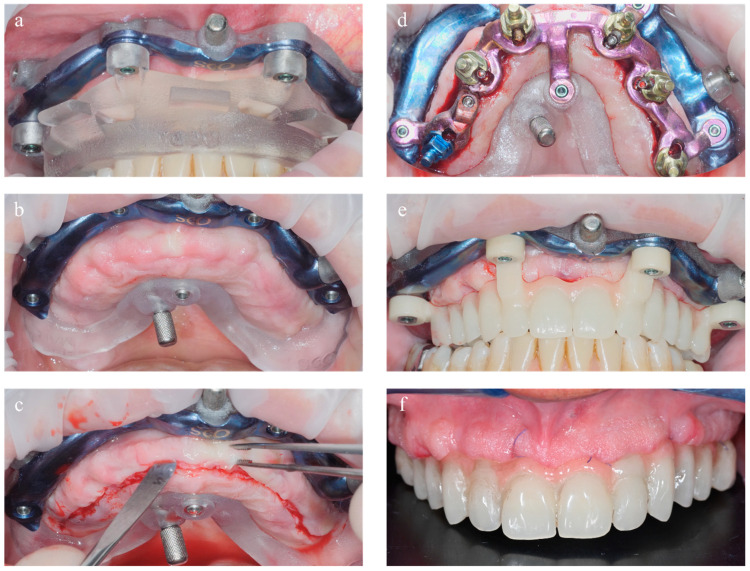
Edentulous surgical workflow. (**a**) Base template connected to the positioning template. (**b**) Base template in position. (**c**) Mucoperiosteal flap. (**d**) Implants’ placement in planned position. (**e**) Provisional prosthesis connected to base template. (**f**) Interim prothesis for immediate loading.

The base template, connected with screws to the mucosa-supported positioning template, was placed in the oral cavity. Proper seating of the mucosa-supported template was achieved by occluding the teeth of the opposite arch in designated areas on the mentioned stent (Figure 4). After verifying the correct placement of the positioning stent through inspection holes, the connected base template was stabilized by means of anchor pins screwed into the bone, buccally and palatally. The mucosa-supported positioning template was then removed, while the base template, anchored to the bone, was left in place until the procedure was completed. In order to preserve the width of the keratinized gingiva, elevation of a mucoperiosteal flap was performed (Figure 8).

The implant stent, completely made of milled grade 5 titanium, was screwed to the base template, and the computer-guided insertion of one-piece implants (FIXO; Oxy Implant Dental System, Biomec, Colico, Italy) was performed. In the posterior regions, to avoid grafting procedures, tilted implants at 17° and 30° were selected. In order to obtain the correct positioning of the fixtures, one-piece implants were inserted with an appropriate implant mounter. This specific mounter was able to compensate for implant angulation during insertion. To verify the correct position of the implants, this specific mounter presented a notch that had to match with the corresponding notch positioned in the sleeve of the surgical template, as planned (Figure 7). Once the two notches matched, the implants were properly seated in apico-coronal and rotational dimensions. At this point, both the implant mounters and the implant template were unscrewed from the base template. All implants achieved an insertion torque ≥35 Ncm. Finally, 6/0 polyglycolic acid sutures were used to seal the flaps.

### 2.3. Prosthetic Restoration

Provisional abutments, adjusted in height according to the virtual waxing, were connected to the fixtures. The interim prosthesis was then screwed to the base template. This prosthesis was made of PMMA reinforced with a metal framework; designed and fabricated according to the diagnostic waxing. The interim prosthesis was relined and intraorally connected to the provisional abutments with a self-polymerizing composite resin (RelyX Ultimate; 3M Italia, Milano, Italy) (Figure 8).

**Figure 9:**
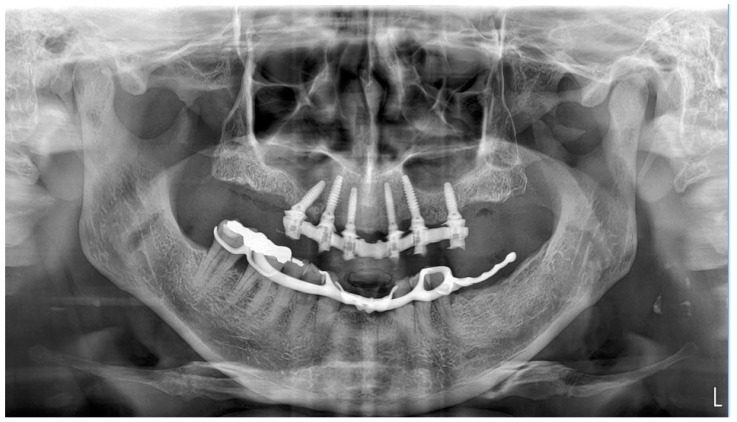
Post-surgical orthopantomography.

After extraoral prosthetic adjustments and polishing, the access holes to the prosthetic screws of the interim abutments were sealed with a photopolymerizable nano-hybrid composite (Tetric EvoFlow; Ivoclar Vivadent, Bolzano, Italy). Occlusal check refinements were made to achieve a proper distribution of mastication forces. A post-operative orthopantomograph was performed for radiological assessment (Figure 9).

## 3. Results

No intraoperative complications, such as profuse bleeding, soft tissue lacerations, template fractures, or implant misplacement with consequent fenestrations or dehiscences occurred during the procedure. All implants achieved a minimum insertion torque of 35 Ncm, so that immediate prosthetic loading could be performed safely. The patient attended follow-up visits each month during the first 3 months of loading. No biological and biomechanical complications were observed. There were no typical signs of peri-implant disease, such as local swelling, soft tissue redness, pain, bleeding upon probing, pus discharge, or the presence of fistulas, within the first 3 months. Conversely, the peri-implant soft tissues appeared to be clinically healthy, characterized by attached keratinized mucosa surrounding the integrated abutments. Post-loading radiographic assessments via orthopantomographs were performed both post-surgery and after 3 months. Bone levels remained radiologically stable, with no detectable signs of remodeling or pathological resorption of the peri-implant crest at the mesial and distal aspects.

## 4. Discussion

The purpose of this technical note is to introduce an innovative approach in computer-guided implantology consisting of stackable templates, sequentially used to simplify and improve the predictability of the workflow, in combination with one-piece implants. Different surgical guides have been developed thus far, depending on the supporting tissue; generally bone, mucosa, or residual teeth. Tooth- and mucosa-supported stents are more precise than bone-supported stents, because inaccuracies in the 3D examination, segmentation, or irregular bone anatomy can significantly alter the position of the latter and, therefore, the precision of implant placement [5,19,20].

Conventional protocols involve the use of an initial tooth- or mucosa-supported guide, which is preliminarily used to create holes for the bone pins. The stent is then removed, any remaining teeth are eventually extracted, and a second implant template is stabilized with fixation pins inserted into the holes made with the previous guide [11]. Sequential removal and insertion of templates and pins may lead to variations in hole direction due to the elastic rebound of the bone [21]. This can dislocate the surgical stents, potentially causing them to assume a position different from the one planned, leading to linear and angular deviations [22]. To overcome this drawback, the method presented here contemplates fixating a base template as a reference that remains in place during the entire procedure. In this way, stabilizing screws are only placed once, thus minimizing errors in repositioning the subsequent stents to the base template, as reported by Granata et al. S [23]. Furthermore, in this protocol, conventional pressure pins were replaced by a new version of screwed pins, improving the stability of the system [21,24]. These pins featured a 1.5 mm thread in the terminal portion, allowing for proper adaptation of the base template to the tissues underneath. It is also worth noting that a fixed template helped the operator switch stents rapidly, reducing the overall operative time. 

As mentioned above, stability is critical in guided implantology. In previous modular protocols, the subsequent components were connected to the base template using ball attachments or magnets [23,24], which could potentially lead to instability during the procedure. On the contrary, in this technical report, screws were used to connect the templates, improving the retention and stability of the entire system.

Another notable difference in comparison to previous stackable templates is that, in this technical note, the rehabilitated patient was entirely edentulous, whereas prior literature predominantly focused on post-extraction rehabilitations [23,24,25].

The material used to fabricate the stackable templates in this protocol deserves some words. Conventional resin-based stereolithographic guides may suffer from deformation or fracturing during implant site preparation [21,26]. Alternatively, stackable templates have been made of cobalt-chrome [24,25] or PMMA in all of their components [23]. In the case shown, to further improve the stability and rigidity of the components, both the base template and the implant insertion stent were fabricated with the inclusion of a milled titanium framework (Figure 6). Titanium enhances stiffness, hence, the risk of deformation and fracture during the drilling sequence was minimized [21]. 

Another limitation of conventional protocols that use closed stents is the inability to properly cool the drills with saline while preparing the implant site. This can lead to an increase in bone temperature, potentially impairing the healing process [27]. This likely results in the formation of scar tissue and consequent fibrointegration of the implant [28]. Conversely, stackable guides were designed to be open, allowing for the direct cooling of the drills during implant bed preparation. 

The quantity and stability of hard and soft tissues play a key role in the long-term success of the rehabilitation [29,30]. In conventional guided implantology, surgical stents often reduce the visibility of the entire surgical field. Moreover, the bulky aspect of traditional templates impedes the management of future peri-implant tissues. In the protocol described herein, the slim and open design of the base template offered an excellent view of the surgical area and allowed for intraoperative management of the tissues underneath. This aspect is particularly important in sites characterized by suboptimal soft tissue thickness [31], where splitting of the keratinized mucosa is mandatory to achieve long-term success. With respect to hard tissues, a resective guide can be connected to the base template, allowing for guided osteoplasty as previously planned in the software, according to the digital waxing. In addition, one-piece implants do not require the use of a bone profiler, which is normally used to create space for prosthetic components in two-piece fixtures. This results in greater bone preservation, also thanks to the reduced thickness of the implant neck, made possible by the lack of an internal connection and a prosthetic screw [18]. In this matter, the absence of an implant-abutment connection also prevents bacterial infiltration, which has been observed in two-piece implants, thereby reducing the likelihood of developing biological complications during follow-up [32]. Interestingly, one-piece implants may also reduce the risk of complications associated with the prosthetic screw. The one used in the present report was characterized by a diameter of 1.8 mm, increasing fracture resistance. 

The one-piece implants inserted with the above-mentioned workflow achieved a minimum insertion torque of 35 Ncm. In the posterior regions, tilted implants at 17° or 30° were placed to avoid grafting procedures. Through digital planning, notches were incorporated into the implant positioning guide so that the axis of the abutment corresponded to the axis of the access holes of the interim prosthesis. Concerning prosthetic advantages, the use of stackable components ensured a more precise position of the temporary restoration during the relining procedures. Indeed, the interim prosthesis was screwed to the base template in order to replicate the position of the framework as accurately as possible according to the digital project. This makes it possible to deliver the prosthesis immediately without having to take impressions [33]. It is also worth mentioning that the increased level of precision achieved by connecting the provisional restoration to the base template reduced the space between the titanium framework and the temporary abutments. Interestingly, no interferences were observed between the prosthesis and the temporary abutments, demonstrating a good level of correspondence between the position of the implant and the digital project. A limitation may be the operator’s experience in both planning and operative phases, as a certain learning curve is required to perform full-digital computer-guided workflows. In addition, the deviation between the planned and achieved implant position using modular templates, which represents a crucial aspect in determining the accuracy of the workflow, currently remains an unsolved issue. Certainly, the correct use of digitalization of all the clinical, surgical, and prosthetic procedures, and the matching of the data into a computerized environment, could take advantage of the combination of collected data to not lose information using classic manual steps and enhance the accuracy of the rehabilitation phases [34]. Last but not least, long-term data are definitely needed to validate the advantages of stackable templates compared to traditional computer-guided workflows.

## 5. Conclusions

The use of stackable templates simplified the operative phases from implant placement to prosthetic loading. The use of a base template served as a fixed reference allowed for the rapid and safe switching of sequential templates, reducing operative time and, ultimately, patient discomfort. The inclusion of a milled titanium framework further increased the rigidity and stability of the system. Open templates also made possible the visualization of the surgical site during the entire procedure, the management of soft tissues, the cooling of the drills during implant bed preparation, and the easy verification of proper implant seating. The ability to screw the temporary prosthesis to the base template during the relining phase constitutes an additional advantage of stackable templates. Additional advantages associated with the use of one-piece implants have been observed in terms of hard tissue preservation, the absence of an implant-abutment connection, and simplified prosthetic phases. All of this, in combination, resulted in adequate primary stability, allowing for immediate loading, optimal soft tissue adaptation around the integrated abutments, and the preservation of marginal bone levels at 3 months from the prosthetic loading.

## Data Availability

Data are available in the article itself.
